# Editorial: Why Vaccines to HIV, HCV, and Malaria Have So Far Failed—Challenges to Developing Vaccines Against Immunoregulating Pathogens

**DOI:** 10.3389/fmicb.2015.01318

**Published:** 2015-11-27

**Authors:** Shuo Li, Magdalena Plebanski, Peter Smooker, Eric J. Gowans

**Affiliations:** ^1^Department of Microbiology, Monash UniversityMelbourne, VIC, Australia; ^2^Swiss Institute of Allergy and Asthma ResearchDavos, Switzerland; ^3^School of Applied Sciences, Health Innovations Research Institute, RMIT UniversityMelbourne, VIC, Australia; ^4^Discipline of Surgery, University of AdelaideAdelaide, SA, Australia

**Keywords:** HIV, HCV, malaria, vaccine, immunity

Together with sanitation, vaccination is one of the most effective life-saving interventions available in the fight against infectious diseases. As you read this issue of Frontier in Microbiology, scientists around the globe are working toward developing vaccines against diverse infectious diseases, allergies, cancer and autoimmune diseases. We believe that every major disease will eventually have its vaccine. However, if we consider major infectious agents, such as human immunodeficiency virus (HIV), hepatitis C virus (HCV), and Malaria, despite many years of effort, billions of dollars spent and countless animal lives sacrificed, no vaccine is available to protect against these infections. How did this happen? What prevents us from being victorious? In this issue of Frontier in Microbiology, we examine why some of these vaccines have failed, collecting reflections from leading researchers in the field.

Some of the key obstacles to vaccine development discussed in this issue include:

1. **The genetic diversity of the target pathogen**. In RNA viruses such as HIV and HCV, the error prone RNA dependent polymerase generates quasispecies (Chanzu and Ondondo, 2014; John and Gaudieri, 2014; Ondondo, 2014). In addition, influenza vaccines need to be reformulated annually, due to antigenic drift (Quinones-Parra et al., 2014). Over half a century of malaria vaccine development, despite awareness of the diversity of natural parasite populations, vaccines that have progressed to human clinical trials have only included a small fraction of the polymorphisms present in endemic regions. In addition to increasing the complexity of the immunogen, antigenic diversity of the organism in different geographic regions has major implications for vaccine efficacy. In this issue, Alyssa Barry and Alicia Arnott discuss the importance of population genetic studies in identifying functionally relevant polymorphisms, and argue that molecular epidemiological surveys are necessary to ensure that the vaccine strain corresponds to the local target parasite populations (Barry and Arnott, 2014). Targeting conserved pathogen antigens may help to overcome diversity, although these regions are often concealed and/or less accessible to immune effectors. Indeed, Chiu and colleagues herein showed that antibody titers to PfRh5 correlated with protection against *Plasmodium falciparum* in clinical trials in PNG (Chiu et al., 2014). Moreover, Quinones-Parra and colleagues also showed that broadly neutralizing antibodies targeting conserved regions, which developed naturally following the 2009 influenza pandemic, provide hints to the nature of the responses a successful vaccine should elicit (Quinones-Parra et al., 2014). As an example of this strategy, Drummer and colleagues proposed that the conserved regions within HCV E2, especially the residues that interact with the virus co-receptor CD81 (Drummer, 2014), may represent an attractive immunogen in a HCV vaccine.
2. **The discrepancy between immunogenicity and protection**. As highlighted in this issue, although many HIV vaccine candidates induce strong T and B cell responses in pre-clinical and Phase-I trials, these responses have thus far failed to correlate with protection in larger scale trials (Chanzu and Ondondo, 2014). The current immunological readouts, such as ELISPOT, intracellular cytokine staining or antibody levels do not appear to be adequate measures that predict vaccine success or failure. New strategies of clinical trial monitoring, such as those depicting immunogenicity from a new angle, or those that dissociate the effects of the vaccine vector from vaccine antigen, may need to be developed and applied. These could include analysis of the T and B cell immune repertoire (Li et al., 2013), which might identify the clonotype response to the vector independent of responses to the immunogen. In addition, whole genome transcription arrays and other recent high throughput assays are likely to provide new and unexpected insights. Overall, new concepts are required to define the best reference or readout of immunogenicity that may in turn predict protective efficacy.
3. **Vector or Immunogen, which one matters?** While an effective vaccine may need to be multivalent, comprising multiple alleles for a given polymorphic antigen, and/or the antigen derived from conserved regions, the delivery vectors are at least as important as the immunogen itself. The vectors modulate innate and adaptive immunity, hopefully enabling the vaccine antigen to elicit the right response (Ondondo, 2014). Prime-boost strategies using plasmid DNA or viral vector prime followed by protein or viral vector boost have been studied extensively. An important lesson was learned from the HIV STEP study, in which a highly immunogenic vaccine actually increased HIV acquisition, presumably due to preexisting immunity to the vector. Of the proposed explanations, a strong response to the vector may have activated CD4^+^ T cells, which are targets for HIV. This seemingly unavoidable paradox highlights the challenges of HIV vaccine development.
4. **The discrepancy between local and systemic responses**. Rafferty and colleagues argue that of the vectors used in HIV vaccine design, viral vectors with mucosal tropism, e.g., adenoviruses and influenza viruses, are particularly interesting, given that genitorectal mucosa is the first site of contact in HIV transmission (Rafferty et al., 2014). Most systemic vaccines do not elicit mucosal responses, and it is uncertain if mucosal delivery of antigen can induce systemic immunity. Cytokines and chemokines have been used as adjuvants to encourage mucosal homing of immune effector cells, such as the “prime-pull” approach in animal models (Rafferty et al., 2014). Difficulties in studying mucosal immune responses, including low cell numbers, sample variation and invasiveness of mucosal sampling means that mucosal immune responses are often not examined in clinical trials, as discussed in this issue (Chanzu and Ondondo, 2014). This is an important area of future clinical trial monitoring and is being addressed. An effective HIV vaccine strategy may need to involve both systemic and mucosal approaches simultaneously. Indeed, women in third world countries share the major burden of HIV infection, and a vaccine that can effectively elicit mucosal immune responses in the female genital tract is more likely to protect women (Rafferty et al., 2014).
5. **Infant vaccination, how much do we know?** On a global scale, millions of infants receive around 20 vaccines during the first year of life, but relatively few studies have examined the development of immunity in this age group. The innate immune system does not reach full capacity until the teenage years, and as adaptive immunity in newborns is intrinsically skewed to a Th2-type, the neonatal and infant immune responses to many vaccines are suboptimal (Ndure and Flanagan, 2014). In addition, the naïve immune repertoire's initial response to a vaccine, which may engage both vaccine antigen-specific and non-specific T and B cells, promoted for example by inflammation via the TNFR2 receptor (Wilson et al., 2015), may play an important role in shaping the repertoire toward subsequent unrelated pathogens. Indeed, growing evidence suggests that vaccines can have heterologous effects, affecting an individual's subsequent responses to unrelated pathogens or vaccines (Flanagan et al., 2011). As vaccines which target major global diseases are eventually likely to be included in childhood vaccination, it is important to understand how vaccines modulate the naïve immune system and the long-term impact of this intervention.
6. **Immune subversion and immunosuppression**. Malaria-infected red blood cells have an amazing capacity to induce FOXP3^+^ expression, a marker of highly suppressive regulatory T cells (Treg), on co-cultured autologous T cells, suggesting that widespread induction *in vivo* would not require direct contact with the parasite (Scholzen et al., 2014). In an exciting mechanistic insight, in this issue, Wykes and colleagues further show a role for PD-1 in malaria-induced loss of T cell function and/or apoptosis (Wykes et al., 2014). While it is not feasible to directly predict viral epitopes recognized by the T cell receptor, Moise and colleagues show that the JanusMatrix algorithm can be applied to achieve this by searching for virus-encoded human homologs, which theoretically can be recognized by Treg (Moise et al., 2014). We also propose that to design improved vaccines we need to better understand how genetic factors such as HLA can affect viral susceptibility. For example, in addition to HIV and HCV which interfere with HLA expression in host immune cells, could HLA intrinsically influence T cell repertoire development before the selection step?

Regardless of the chances of developing a prophylactic vaccine for every disease, the world needs vaccines to reduce current disease burdens and save lives. How to effectively mobilize innate immunity may be another focus for future vaccine design. Learning from our mistakes and understanding our limitations will help us in our ongoing battle against pathogens.

## Author contributions

SL wrote the manuscript; MP, PS, and EG helped with opinions, suggestions, and editing.

### Conflict of interest statement

The authors declare that the research was conducted in the absence of any commercial or financial relationships that could be construed as a potential conflict of interest.
